# Retrospective assessment of rapid outbreak investigation for gastrointestinal diseases using only cases and background exposure data

**DOI:** 10.1017/S0950268820000527

**Published:** 2020-02-21

**Authors:** G. Kafatos, P. Mook, A. Charlett, E. Rees, R. Elson, T. Inns, S. Kanagarajah, N. J. Andrews

**Affiliations:** 1 Statistics, Economics and Modelling Department, National Infection Service, Public Health England, London, UK; 2 Field Epidemiology Service South East and London, National Infection Service, Public Health England, London, UK; 3 Warwick Medical School, University of Warwick, Coventry, UK; 4 National Infection Service, Public Health England, London, UK; 5 Field Epidemiology North West, National Infection Service, Public Health England, Liverpool, UK

**Keywords:** Case-background, epidemiology, gastrointestinal, outbreak, trawling

## Abstract

For outbreaks of gastrointestinal disease, rapid identification of the source is crucial to enable public health intervention and prevent further cases. Outbreak investigation comprises analyses of exposure information from cases and, if required, undertaking analytical epidemiological studies. Hypothesis generation has been reliant on empirical knowledge of exposures historically associated with a given pathogen. Epidemiology studies are resource-intensive and prone to bias, one of the reasons being the difficulties in recruiting appropriate controls. For this paper, the information from cases was compared against pre-defined background exposure information. As exemplars, three past outbreaks were used, one of common and two of rare exposures. Information from historical case trawling questionnaires was used to define background exposure having removed any exposures implicated with the outbreak. The case-background approach showed good sensitivity and specificity, identifying correctly all outbreak-related exposures. One additional exposure related to a retailer was identified and four food items where all cases had been exposed. In conclusion, the case-background method, a development of the case-case design, can be used to assist with hypothesis generation or when a case-control study may not be possible to carry out.

## Introduction

The burden of infectious intestinal diseases (IID) is generally underestimated by national surveillance systems [1]. A population-based study in the United Kingdom (UK) estimated the annual burden of IID as 17 million sporadic cases (not directly related to an outbreak) [2]. In addition to this, over 800 local and national outbreaks affecting ~13 000 people were reported to Public Health England (PHE) between 2000 and 2011 [3]. The overall cost of IID in the UK has been estimated as £743 m in 1994/95 prices which is equivalent to £1.4b in 2018 prices [4, 5].

The definition of an outbreak is two or more cases of the same disease that share an epidemiological link or where the observed number of cases exceeds the expected number. Outbreaks of IID in the UK are detected using an exceedance algorithm [6]. Potential outbreaks of bacterial pathogens have been identified using whole genome sequencing since its introduction as a routine typing tool since 2014. Various combinations of descriptive, microbiological or (descriptive or analytic) epidemiological evidence may be provided [7]. Additional evidence can also be obtained from product tracing [8].

The majority of the IID outbreaks reported to PHE were investigated at a local level and some that crossed regional boundaries were investigated at a national level. For example, of the 391 outbreaks caused by *Salmonella enterica* 52 were classified as national outbreaks [3]. For ongoing IID outbreaks it is important that the source or vehicle of infection is identified as rapidly as possible to implement public health intervention to prevent future cases. There are numerous examples of successful public health interventions, including fast food premises closure for a large *Salmonella* outbreak that took place in England in 2005, or raising public awareness for the correct preparation of raw bean sprouts for an *S.* Bareilly outbreak in the UK in 2010 [9, 10].

Analytical epidemiological studies to investigate IID outbreaks obtain exposure information using bespoke questionnaires. The purpose being to test specific hypotheses, so it is important that clear hypotheses are generated prior to the initiation of any such study [11]. For disseminated outbreaks, hypotheses are often generated by administering comprehensive trawling questionnaires (so named as it casts a wide net) which include an in-depth examination of common exposures from a sample of recent cases. The aim is to uncover common exposures that may be causal, in order to conduct a focused analytical epidemiological study [12]. In practice, at the national IID team in PHE, exposures are being assessed in a semi-subjective manner guided by the percentage of cases exposed, with cut-offs of 60% for general exposure (e.g. drank milk) and 70% for specific exposures (e.g. drank milk from supermarket A) frequently used. A common PHE and Health Protection Scotland trawling questionnaire template can be found on the UK government website [13].

Community outbreaks where cases are clustered in time or area are often investigated using descriptive analysis [12]. For ~40% of outbreaks where the evidence base used to support conclusions is known, descriptive epidemiology was carried out based on analyses of case information [7]. Descriptive analysis alone is often used when the number of cases is insufficient to perform an analytical study. Analytical epidemiological studies are conducted as a part of the investigation (most often of case-control design) of national or sub-national outbreaks when the outbreak source is not apparent following descriptive analyses [3].

For a minority of the outbreaks investigated by PHE no clear vehicle is being identified [3]. There could be several reasons for the failure of these studies to identify the outbreak vehicle. Reasons may be an insufficient sample size, which is often attributed to difficulties in recruiting controls, food cross-contamination in the kitchen or asymptomatic cases wrongly classified as controls [14, 15]. Moreover, the contaminated food can be very common (e.g. chicken) and therefore, it may be difficult to determine whether cases consume disproportionally high quantity of it [3]. Another reason could be the omission of the true outbreak source from the hypotheses to be tested. An example of this was the 2011 entero-haemorrhagic *E. coli* outbreak in Germany where the outbreak source was an often-unrecognised exposure (fenugreek sprouts) [16, 17]. Initial studies failed to identify the true cause and pointed to confounded exposures of raw tomatoes, cucumbers and leafy salads. While the consumption of sprouts has been previously implicated in other outbreaks, only 25% of cases in the trawling interviews recalled this exposure [16–18].

This paper aims to describe a simple yet robust method for quantifying background exposure information reported by cases from historic outbreaks and comparing it against exposures reported by cases during an outbreak. This case-background method is mainly intended in guiding hypotheses generation but can also be used as an additional method when a case-control study may not be possible to carry out.

## Methods

### Case-background method using individual-level background exposure data

Background exposure information is often available from previous outbreak investigations where detailed individual exposure information has been obtained from cases using trawling questionnaires. This background exposure information is then used to provide comparative exposure odds.

The odds ratio (OR_CB_) is calculated by dividing the information from the case-questionnaire (odds_case_) by the one from the background exposure (odds_BE_):1
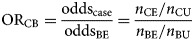
Assume *n*_CE_ and *n*_CU_ are the exposed and unexposed cases, respectively and *n*_BE_ and *n*_BU_ are the exposed and unexposed background ‘controls’, respectively. The calculation of the confidence interval (CI) was performed using the formula described by Bland and Altman [19].

Looking as an example at the 2011 entero-haemorrhagic *E. coli* outbreak in Germany, the consumption of sprouts was mentioned by only 25% of the initial cases interviewed using a trawling questionnaire [16]. As this was well below the 50% cut-off used for rarer exposures, sprouts were initially not suspected as the outbreak vehicle and were not considered for the initial case-control study. Using the case-background approach and assuming that (i) the initial number of cases considered was 20 and (ii) consumption of sprouts was confirmed for two out of 40 background exposure questionnaires, the resulting odds ratio is the following:
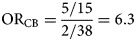


An odds ratio of 6.3 with a 95% CI of 1.1–36.3 suggests a link between sprouts and being a case, worthy of further investigation.

### Case-background method with no individual-level data for background exposure

#### Measure of association

**Fig. 1.**
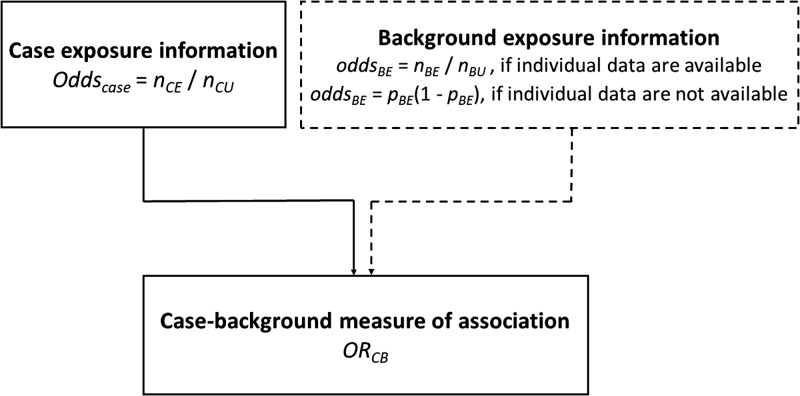
Case-background measures of association.

An alternative approach to that described above is to use aggregate summary information from sources such as a literature review, existing population-based surveys or consumer websites. There, the background exposure information may simply be expressed as a fraction *p*_BE_. To estimate OR_CB_, odds_BE_ = *p*_BE_/(1 − *p*_BE_) needs to be substituted in Formula (1).2
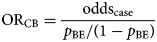
(Fig. 1).

For the 2011 *E. coli* outbreak in Germany assuming the consumption of sprouts in the general population in the relevant time-period is 5%, the resulting odds ratio would be:
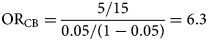


#### Bootstrap confidence intervals

Assuming no information is provided other than the fraction *p*_BE_ (unknown numerator and denominator), the following approach is proposed to derive 95% Bootstrap confidence intervals (CI_CB_) around the point estimate, OR_CB_:

1. Assign ‘proxy’ uncertainty intervals UI_BE_ = (*α*, *β*) around the background exposure estimate *p*_BE_.
2. Generate *i* = 1000 simulations based on uniform distribution 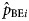
~ *U*(*α*, *β*) (note that other type of distributions can be used).
3. Substitute 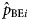 in Formula (2) to obtain OR_CB*i*_. The 95% CI_CB_ for OR_CB_ can be defined as the 2.5^th^ and the 97.5^th^ percentiles of OR_CB*i*_.

Using the same Germany *E. coli* example as above:

1. Suppose the uncertainty intervals around a percentage of background exposure of 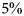 are between 2.5% and 7.5% (UI_BE_ = (2.5, 7.5)).
2. Generate 1000 simulations from 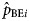 ~ *U*(0.025, 0.075)
3. Substituting in Formula (2), for OR_CB_ = 6.3, the 95% CI_CB_ is (4.20, 12.3).

### An application of the case-background method

#### Background exposure data

In the examples presented in this paper, the population background exposure was quantified based on information from four outbreak trawling questionnaires in which the exposure associated with the outbreak was conclusively known. These outbreaks occurred in the United Kingdom between 2008 and 2015 and included a total of 72 trawling questionnaires [20–23]. Sixty-seven percent of the participants were females and the median age was 43 years (interquartile range (IQR): (22, 56)) (Table 1).

**Table 1.** Summary of outbreaks used to generate background exposure

Outbreak pathogen	Year	Outbreak vehicle	Region	Reference	Trawling questionnaire participant characteristics		
					Number of trawls	% female	Age (median, IQR)
*S.* Enteritidis PT59	2015	Eggs	North-West	[22]	11	73%	40 (22, 54)
*VTEC* PT8	2010	Raw potato and leeks	Nationwide	[23]	22	80%	39 (12, 59)
*S.* Mikawasima	2013	Chicken	Scotland, West Midlands and Wales	[21]	27	48%	44 (29, 57)
*S.* Typhimurium PT U320	2008	Ready-to-eat salad	England	[20]	12	82%	27 (9, 49)

Variables related to exposure were set to missing for each specific set of trawling questionnaires. For example, for the *S.* Enteritidis PT59 example, where eggs were identified as the vehicle, any variables related to egg consumption (such as omelettes, egg sandwich, egg salads, quiches and souffles) were removed. The remaining exposures were coded as binary yes/no. Information on symptoms, case contacts and travel history were discarded. Additional constructed exposure variables were created where appropriate.

#### Setting up the case questionnaires

Three outbreaks (two of rare [24, 25] and one of common [26, 27] exposures) were selected as examples to test the case-background methodology (Table 2). The information was extracted from case-questionnaires. Two of these questionnaires had been used as part of case-control studies [24, 25]. An analytical study had not been carried out for the third (*S.* Senftenberg) but some trawling information had been collected [26, 27].

**Table 2.** Summary of outbreaks used as examples to demonstrate the case-background method

Outbreak pathogen	Year	Outbreak vehicle	Region	Reference	Number of cases
*S.* Senftenberg	2007	Basil	England and Wales	[26, 27]	20
*S.* Enteritidis PT8	2015	Feeder mice for reptiles	Nationwide	[25]	26
*S.* Typhimurium DT 191A	2009	Feeder mice for reptiles	England and Wales	[24]	21

The data manipulation was carried out in the same way as for the trawling questionnaires, however, the information related to the implicated exposure vehicle was retained in the analysis. For each outbreak, exposures that were common to both case and control datasets were used for the analysis.

#### Analysis

For the analysis described in this paper, any exposures with an odds ratio (OR_BE_) greater or equal to 2 and with a lower limit of confidence interval CI_CB_ above 1 were considered as potential causal exposure.

For zeros in the numerators or denominators in Formula (1) and Formula (2) (i.e. all cases or ‘controls’ exposed or unexposed), an arbitrary value between 0 and 1 (i.e. 0.5) was added for each 2 × 2 cell. For *p*_BE_ = 1 then *p*_BE_ was replaced by 0.99. No CIs were provided for these cases.

The analysis was carried out using Stata statistical software (StataCorp. 2015. Stata Statistical Software: Release 14. College Station, TX: StataCorp LP).

## Results

A total of 560 binary variables were used as background exposure information. For the great majority of these variables (*n* = 480; 86%), the proportion of individuals exposed was less than 50% (Table 3).

**Table 3.** Number of variables used for the background exposure information by proportions of ‘controls’ exposed (i.e. <10% and ≥90% denote very rare and common exposures, respectively)

Proportion of ‘controls’ exposed	Number of exposure variables
<10%	309
10%–<30%	129
30%–<50%	42
50%–<70%	40
70%–<90%	27
≥90%	13
Total	560

### Case-background method using individual-level background exposure data

#### *S.* Senftenberg outbreak

**Fig. 2.**
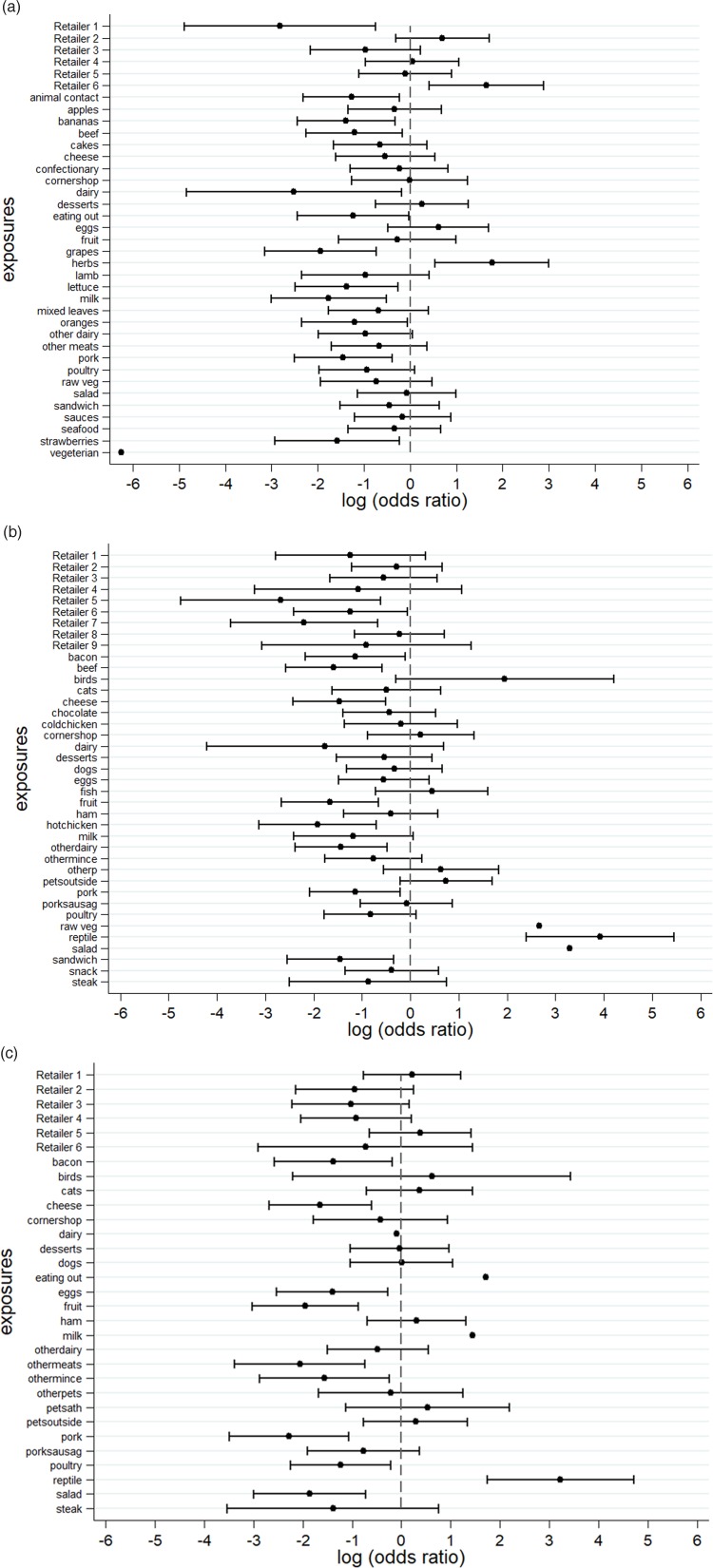
Case-background method using individual-level background exposure data. (a) *S.* Senftenberg, (b) *S.* Enteritidis PT8 and (c) *S.* Typhimurium DT19A. *Note*: No CIs were provided if all cases or individuals within the background population were exposed or unexposed (i.e. zeros in numerator or denominator).

**Fig. 3.**
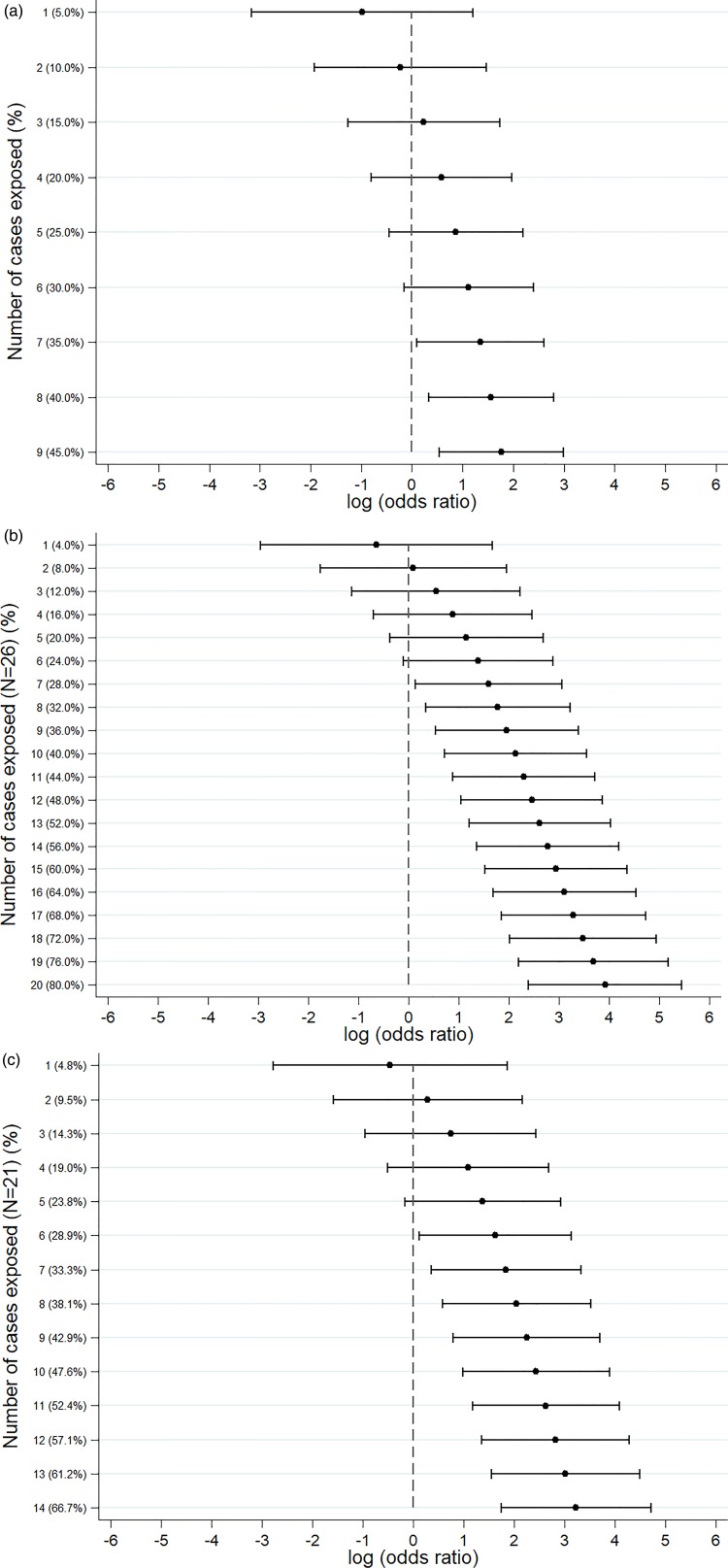
Sensitivity analysis showing the log(odds ratio) for different numbers of cases exposed. (a) *S.* Senftenberg – consumption of any herbs. (b) *S.* Enteritidis PT8 – feeder mice. (c) *S.* Typhimurium DT19A – feeder mice.

An *S.* Senftenberg outbreak was used as an example that took place during 2007. This was an international outbreak with cases reported in England and Wales [26, 27]. The case-background method was applied on 37 exposure variables. ‘Retailer 6’ (OR_CB_ 5.2; 95% CI_CB_ 1.5–18.1) and ‘consumption of any type of herbs’ (OR_CB_ 5.9; 95% CI_CB_ 1.7, 20.0) had CIs above 1 (Fig. 2a). Nine out of 20 cases (45%) included in the analysis indicated that they had consumed any type of herbs 5 days prior to the occurrence of symptoms. The minimum number of cases with which the exposure would have been flagged was seven cases (35%) with OR_CB_ 3.9 (95% CI_CB_ 1.1–13.5) (Fig. 3a).

#### *S.* Enteritidis PT8 outbreak

Over 4 years (2012–2015) a UK nationwide *S.* Enteritidis outbreak was investigated by PHE [25]. Information from 39 exposure variables was included. Of these, ‘having a reptile as a domestic pet’ (OR_CB_ 50.7; 95% CI_CB_ 11.0–234.1) had the strongest association. The items ‘consumption of salad’ (OR_CB_ 26.8) and ‘consumption of raw vegetables’ (OR_CB_ 14.3) also had strong association as all 26 cases questioned had been exposed (Fig. 2b). Of the 25 cases interviewed, 20 (80%) indicated that their family kept reptiles at home as domestic pets whereas five said that this was not the case. If instead of 20, seven cases (28%) had confirmed keeping reptiles as domestic pets the association would have been OR_CB_ 4.9 (95% CI_CB_ 1.1–21.3) (Fig. 3b).

#### *S.* Typhimurium DT19A outbreak

In 2009, an *S.* Typhimurium outbreak occurred in England and Wales [24]. Of the 31 exposures included in the analysis, ‘having a reptile as a domestic pet’ had OR_CB_ 25.3 (95% CI_CB_ 5.7–111.8). For the items ‘consumption of milk’ (OR_CB_ 4.3; *n*_CE_ = 21) and ‘eating out’ (OR_CB_ 5.5; *n*_CE_ = 13), all cases questioned had been exposed (Fig. 2c). Fourteen out of 21 cases (66.6%) indicated that their family keeps reptiles at home as domestic pets. For six out of 21 cases (28.6%) the association would still have been flagged (OR_CB_ = 5.1 (95% CI_CB_ 1.1–22.9)) (Fig. 3c).

### Case-background method using ‘proportion of exposure’ to define background exposure

The examples shown used routinely available individual background exposure data collected using questionnaires. The analysis was re-run assuming that only the proportions of background exposure *p*_EB_ and corresponding ‘proxy’ uncertainty intervals (set up as UI_BE_ = 40%) were provided. The same exposures as above were found to be associated with the outbreaks. The number of ‘controls’ and cases by exposure variable together with the OR_CB_ and CI_CB_ are given in Supplement Table 1.

## Discussion

For the purposes of this paper, background exposure information came from trawling questionnaires that were stripped of any outbreak-related exposures and compared with three exemplar outbreak case datasets. In all these examples, the true source of the outbreak was identified successfully. Five additional exposures were flagged up (four of them had all cases exposed plus a retailer which could be genuinely associated with the source), which demonstrates the specificity of the method. The analysis was re-run assuming unknown total and exposed numbers for the background (only the proportion exposed, *p*_BE_, was known) which resulted in identifying the same exposures.

Two of the outbreaks used as examples had uncommon exposure (feeder mice for reptiles). The third was common exposure, however, herbs are food items whose consumption is not well recalled in general. None of the examples used had an outbreak source of common exposure such as consumption of eggs or chicken. Looking at the recent outbreak investigations with identifiable egg vehicles, a *S.* Heidelberg outbreak investigation associated with in-flight catering identified two sources: milk tart (OR 10.2; 95% CI 2.0–52.8) and an egg dish/omelette (OR 6.0; 95% CI 1.3–27.3) [28]. Applying the case-background formula on the two sources identified, the corresponding OR_CB_ would have been 41.6 (95% CI 9.3–185.2) and 9.3 (95% CI 2.7–32.3) for the milk pudding and omelette, respectively. Looking at a *Campylobacter jejuni* outbreak that occurred in an Australian university college, the univariate analysis identified chicken liver pȃté (RR 3.6; 95% CI 2.0–6.5) as the likely cause of infection [29]. Applying the case-background formula to the exposure identified, resulted in OR_CB_ 7.1 (95% CI 1.6–31.7).

The case-background method can also be applied using background exposure not captured by a questionnaire i.e. when patient-level data are not available. This allows for background population exposure to be extracted from consumer websites (web scraping) or population survey data [30]. Alternatively, expert consensus based on the investigators knowledge and experience can be translated into a ‘proportion of population exposed’ and corresponding uncertainty intervals. However, before such data sources can be used as background exposure information it is important to fully understand the study design and data collection methods and therefore, any potential biases related to recalled exposures.

A number of methods have been proposed in the past that include comparing cases against different types of information for ‘controls’ such as market research panels using online questionnaires [31, 32], case-case study designs [14, 33, 34] and a simulation-type approach for generating ‘control’ information [35]. An attempt has been made in the Netherlands to collect background population exposure via an infectious disease repeated electronic survey that can then be used in outbreak investigations [36]. This paper proposes a similar case-background method approach that compares the information from cases against routinely available background exposure information. In the examples shown background exposure was built based on four case trawling questionnaires after the variables associated with being a case were removed. Although case trawling questionnaires were used herein, ideally the background information should be obtained from trawling questionnaires carried out on healthy controls. Ideally, these controls should be collected from a representative sample of the population cases were originated from. In this way, some of the limitations associated with the case-case design and the bias arising from using cases are being eliminated [34]. Once this background information has been set up, it can be used for multiple outbreak investigation studies.

Despite the successful demonstration of the case-background method using the examples above, caution is advised when used in practice. The main assumption of this method is that the population used to generate the background information is the same population cases are samples from. Therefore, caution is required when there are temporal, spatial or demographic differences. Moreover, it is important to ensure that the ‘exposure period’ is defined in the same way between the case and the control population (for the trawling questionnaire interviews included here, the ‘controls’ were asked to recall exposures for the last 5 days). National consumption surveys or alternative sampled surveys could be used to obtain robust background exposure information. These surveys should account for factors such as different population groups or seasonality effects. They should be repeated every few years (say 5–7 years) to incorporate changing population behaviours such as food preferences. If data from cases are used as background exposure, it might be prudent to remove food exposures that during the original analysis were identified as having an association with illness confounded by the identified outbreak vehicle because the distribution of these exposures would be systematically different from that of the underlying population. Moreover, the case-background methodology can be used to account for different types of underlying distributions and CI widths. The method can also be extended to multivariable analysis. For example, the proportion of females in the background data was 66.7%. For the *S.* Typhimurium DT19A outbreak the proportion of females was 57.1%. After including gender in a multivariable logistic regression model, the adjusted odds ratio for ‘having a reptile as a domestic pet’ marginally increased to OR_CB_ 27.3 (5.9–127.8). Ultimately, the successful application of the case-background method will be dependent on routinely available, contemporaneous population background exposure data.

Currently, hypothesis generation relies on empirical knowledge of exposures historically associated with a given pathogen. Herein, we propose a case-background method where the background information is pre-defined either from past case trawling questionnaires or alternatively from carefully selected controls. This information can be used for multiple outbreak investigation studies as part of hypothesis generation or as an additional method when a case-control study may not be possible to carry out. In its simpler form, the method proposed does not require specialist statistical knowledge or software and can be easily employed in different scenarios such as by field epidemiologists. The case-background method can assist with optimising the design of analytical gastrointestinal outbreaks studies which might increase the speed with which public health interventions can be deployed.

## Supplementary material

For supplementary material accompanying this paper visit https://doi.org/10.1017/S0950268820000527. click here to view supplementary material
