# Supplementary material for: Retrospective assessment of rapid outbreak investigation for gastrointestinal diseases using only cases and background exposure data
Source: Epidemiol Infect. 2020 Feb 21;148:e60. doi: 10.1017/S0950268820000527 (PMC7078580; doi:10.1017/S0950268820000527)
Supplement: Supplementary file 1 [file S0950268820000527sup001.docx]

**Supplement Table 1**

(a) *S.* Senftenberg

| **Exposure** | **Total number of cases** | **Number of cases exposed** | **Total number of “controls” (background exposure)** | **Number of “controls” exposed** | **OR** | **known individual-level data 95% CI** | **unknown individual-level data 95% CI** |
| --- | --- | --- | --- | --- | --- | --- | --- |
| Retailer 1 | 20 | 1 | 66 | 31 | 0.1 | (0.0, 0.5) | (0.0, 0.1) |
| Retailer 2 | 20 | 11 | 66 | 25 | 2.0 | (0.7, 5.5) | (0.9, 5.2) |
| Retailer 3 | 20 | 4 | 68 | 27 | 0.4 | (0.1, 1.3) | (0.2, 1.0) |
| Retailer 4 | 20 | 9 | 66 | 29 | 1.0 | (0.4, 2.9) | (0.5, 2.4) |
| Retailer 5 | 20 | 11 | 66 | 38 | 0.9 | (0.3, 2.5) | (0.4, 1.9) |
| Retailer 6 | 20 | 7 | 64 | 6 | 5.2 | (1.5, 18.1) | (1.3, 66.4) |
| animal contact | 20 | 7 | 70 | 46 | 0.3 | (0.1, 0.8) | (0.1, 0.6) |
| apples | 20 | 10 | 60 | 35 | 0.7 | (0.3, 2.0) | (0.3, 1.5) |
| bananas | 20 | 9 | 64 | 49 | 0.3 | (0.1, 0.7) | (0.0, 0.6) |
| beef | 20 | 7 | 70 | 45 | 0.3 | (0.1, 0.8) | (0.1, 0.6) |
| cakes | 20 | 10 | 70 | 46 | 0.5 | (0.2, 1.4) | (0.2, 1.1) |
| cheese | 20 | 13 | 67 | 51 | 0.6 | (0.2, 1.7) | (0.1, 1.4) |
| confectionary | 20 | 13 | 67 | 47 | 0.8 | (0.3, 2.3) | (0.2, 1.8) |
| corner shop | 20 | 4 | 64 | 13 | 1.0 | (0.3, 3.4) | (0.4, 17.5) |
| dairy | 20 | 17 | 71 | 70 | 0.1 | (0.0, 0.8) | (0.0, 1.5) |
| desserts | 20 | 9 | 72 | 28 | 1.3 | (0.5, 3.5) | (0.6, 3.3) |
| eating out | 20 | 12 | 43 | 36 | 0.3 | (0.1, 1.0) | (0.0, 0.8) |
| eggs | 20 | 14 | 59 | 33 | 1.8 | (0.6, 5.4) | (0.8, 4.0) |
| fruit | 20 | 16 | 69 | 58 | 0.8 | (0.2, 2.7) | (0.0, 2.1) |
| grapes | 20 | 4 | 63 | 40 | 0.1 | (0.0, 0.5) | (0.1, 0.3) |
| herbs | 20 | 9 | 49 | 6 | 5.9 | (1.7, 20) | (1.8, 91.8) |
| lamb | 20 | 3 | 44 | 14 | 0.4 | (0.1, 1.5) | (0.2, 1.2) |
| lettuce | 20 | 7 | 44 | 30 | 0.3 | (0.1, 0.8) | (0.1, 0.6) |
| milk | 20 | 13 | 71 | 65 | 0.2 | (0.0, 0.6) | (0.0, 0.7) |
| mixed leaves | 20 | 8 | 42 | 24 | 0.5 | (0.2, 1.5) | (0.2, 1.1) |
| oranges | 20 | 5 | 57 | 30 | 0.3 | (0.1, 0.9) | (0.1, 0.7) |
| other dairy | 20 | 10 | 69 | 50 | 0.4 | (0.1, 1.1) | (0.1, 0.9) |
| other meats | 20 | 8 | 60 | 34 | 0.5 | (0.2, 1.4) | (0.2, 1.1) |
| pork | 20 | 7 | 66 | 46 | 0.2 | (0.1, 0.7) | (0.1, 0.5) |
| poultry | 20 | 11 | 70 | 53 | 0.4 | (0.1, 1.1) | (0.1, 0.9) |
| raw veg | 20 | 13 | 39 | 31 | 0.5 | (0.1, 1.6) | (0.0, 1.2) |
| salad | 20 | 13 | 60 | 40 | 0.9 | (0.3, 2.7) | (0.3, 2.0) |
| sandwich | 20 | 9 | 41 | 23 | 0.6 | (0.2, 1.9) | (0.3, 1.4) |
| sauces | 20 | 10 | 48 | 26 | 0.8 | (0.3, 2.4) | (0.4, 1.8) |
| seafood | 20 | 9 | 69 | 37 | 0.7 | (0.3, 1.9) | (0.3, 1.5) |
| strawberries | 20 | 3 | 52 | 24 | 0.2 | (0.1, 0.8) | (0.1, 0.5) |
| vegetarian | 20 | 3 | 51 | 51 | 0.0 |  |  |

(b) *S.* Enteritidis PT8

| **Exposure** | **Total number of cases** | **Number of cases exposed** | **Total number of “controls” (background exposure)** | **Number of “controls” exposed** | **OR** | **known individual-level data 95% CI** | **unknown individual-level data 95% CI** |
| --- | --- | --- | --- | --- | --- | --- | --- |
| Retailer 1 | 25 | 2 | 65 | 15 | 0.3 | (0.1, 1.4) | (0.1, 2.0) |
| Retailer 2 | 25 | 10 | 66 | 31 | 0.8 | (0.3, 1.9) | (0.3, 1.7) |
| Retailer 3 | 25 | 5 | 66 | 20 | 0.6 | (0.2, 1.7) | (0.3, 1.9) |
| Retailer 4 | 25 | 1 | 64 | 7 | 0.3 | (0.0, 2.9) | (0.1, 4.9) |
| Retailer 5 | 25 | 1 | 66 | 25 | 0.1 | (0.0, 0.5) | (0.0, 0.2) |
| Retailer 6 | 25 | 4 | 68 | 27 | 0.3 | (0.1, 0.9) | (0.1, 0.7) |
| Retailer 7 | 25 | 2 | 66 | 29 | 0.1 | (0.0, 0.5) | (0.1, 0.3) |
| Retailer 8 | 25 | 13 | 66 | 38 | 0.8 | (0.3, 2.0) | (0.3, 1.7) |
| Retailer 9 | 25 | 1 | 64 | 6 | 0.4 | (0.0, 3.5) | (0.1, 5.1) |
| bacon | 26 | 6 | 62 | 30 | 0.3 | (0.1, 0.9) | (0.1, 0.7) |
| beef | 26 | 7 | 70 | 45 | 0.2 | (0.1, 0.6) | (0.1, 0.4) |
| birds | 25 | 4 | 38 | 1 | 7.0 | (0.7, 67.3) | (0.7, 30.5) |
| cats | 25 | 6 | 41 | 14 | 0.6 | (0.2, 1.9) | (0.3, 1.8) |
| cheese | 26 | 11 | 67 | 51 | 0.2 | (0.1, 0.6) | (0.0, 0.5) |
| chocolate | 26 | 16 | 66 | 47 | 0.6 | (0.2, 1.7) | (0.2, 1.5) |
| cold chicken | 26 | 7 | 29 | 9 | 0.8 | (0.3, 2.6) | (0.4, 2.7) |
| corner shop | 25 | 6 | 64 | 13 | 1.2 | (0.4, 3.7) | (0.5, 22.1) |
| dairy | 26 | 24 | 71 | 70 | 0.2 | (0.0, 2.0) | (0.1, 3.2) |
| desserts | 26 | 7 | 72 | 28 | 0.6 | (0.2, 1.6) | (0.3, 1.5) |
| dogs | 25 | 11 | 44 | 23 | 0.7 | (0.3, 1.9) | (0.3, 1.6) |
| eggs | 26 | 11 | 59 | 33 | 0.6 | (0.2, 1.5) | (0.2, 1.2) |
| fish | 25 | 7 | 40 | 8 | 1.6 | (0.5, 5.0) | (0.6, 35.1) |
| fruit | 26 | 13 | 69 | 58 | 0.2 | (0.1, 0.5) | (0.0, 0.5) |
| ham | 26 | 8 | 60 | 24 | 0.7 | (0.3, 1.8) | (0.3, 1.7) |
| hot chicken | 26 | 13 | 39 | 34 | 0.1 | (0.0, 0.5) | (0.0, 0.5) |
| milk | 26 | 20 | 71 | 65 | 0.3 | (0.1, 1.1) | (0.0, 1.3) |
| other dairy | 26 | 10 | 69 | 50 | 0.2 | (0.1, 0.6) | (0.1, 0.5) |
| other mince | 26 | 7 | 61 | 27 | 0.5 | (0.2, 1.3) | (0.2, 1.1) |
| otherp | 25 | 7 | 41 | 7 | 1.9 | (0.6, 6.2) | (0.7, 37.9) |
| pets outside | 25 | 11 | 66 | 18 | 2.1 | (0.8, 5.5) | (0.9, 8.6) |
| pork | 26 | 11 | 66 | 46 | 0.3 | (0.1, 0.8) | (0.1, 0.7) |
| pork sausage | 26 | 10 | 57 | 23 | 0.9 | (0.4, 2.4) | (0.4, 2.3) |
| poultry | 26 | 15 | 70 | 53 | 0.4 | (0.2, 1.1) | (0.1, 1.0) |
| raw veg | 26 | 26 | 39 | 31 | 14.3 |  |  |
| reptile | 25 | 20 | 41 | 3 | 50.7 | (11, 234.1) | (11.1, 530.4) |
| salad | 26 | 26 | 60 | 40 | 26.8 |  |  |
| sandwich | 26 | 6 | 41 | 23 | 0.2 | (0.1, 0.7) | (0.1, 0.5) |
| snack | 26 | 14 | 49 | 31 | 0.7 | (0.3, 1.8) | (0.3, 1.5) |
| steak | 26 | 2 | 48 | 8 | 0.4 | (0.1, 2.1) | (0.2, 8.2) |

(c) *S.* Typhimurium DT19A

| **Exposure** | **Total number of cases** | **Number of cases exposed** | **Total number of “controls” (background exposure)** | **Number of “controls” exposed** | **OR** | **known individual-level data 95% CI** | **unknown individual-level data 95% CI** |
| --- | --- | --- | --- | --- | --- | --- | --- |
| Retailer 1 | 21 | 11 | 66 | 31 | 1.2 | (0.5, 3.3) | (0.6, 2.8) |
| Retailer 2 | 21 | 4 | 66 | 25 | 0.4 | (0.1, 1.3) | (0.2, 1.0) |
| Retailer 3 | 21 | 4 | 68 | 27 | 0.4 | (0.1, 1.2) | (0.2, 0.9) |
| Retailer 4 | 21 | 5 | 66 | 29 | 0.4 | (0.1, 1.2) | (0.2, 0.9) |
| Retailer 5 | 21 | 14 | 66 | 38 | 1.5 | (0.5, 4.1) | (0.6, 3.2) |
| Retailer 6 | 21 | 1 | 64 | 6 | 0.5 | (0.1, 4.3) | (0.1, 6.2) |
| bacon | 21 | 4 | 62 | 30 | 0.3 | (0.1, 0.8) | (0.1, 0.6) |
| birds | 21 | 1 | 38 | 1 | 1.9 | (0.1, 31.2) | (0.2, 8.0) |
| cats | 21 | 9 | 41 | 14 | 1.4 | (0.5, 4.3) | (0.7, 4.2) |
| cheese | 21 | 8 | 67 | 51 | 0.2 | (0.1, 0.5) | (0.0, 0.5) |
| corner shop | 21 | 3 | 64 | 13 | 0.7 | (0.2, 2.6) | (0.3, 11.7) |
| dairy | 21 | 21 | 71 | 70 | 0.9 |  |  |
| desserts | 21 | 8 | 72 | 28 | 1.0 | (0.4, 2.6) | (0.5, 2.5) |
| dogs | 21 | 11 | 44 | 23 | 1.0 | (0.4, 2.8) | (0.4, 2.2) |
| eating out | 13 | 13 | 43 | 36 | 5.5 |  |  |
| eggs | 21 | 5 | 59 | 33 | 0.2 | (0.1, 0.8) | (0.1, 0.5) |
| fruit | 21 | 9 | 69 | 58 | 0.1 | (0.0, 0.4) | (0.0, 0.4) |
| ham | 21 | 10 | 60 | 24 | 1.4 | (0.5, 3.7) | (0.6, 3.4) |
| milk | 21 | 21 | 71 | 65 | 4.3 |  |  |
| other dairy | 21 | 13 | 69 | 50 | 0.6 | (0.2, 1.7) | (0.2, 1.4) |
| other meats | 21 | 3 | 60 | 34 | 0.1 | (0.0, 0.5) | (0.1, 0.3) |
| other mince | 21 | 3 | 61 | 27 | 0.2 | (0.1, 0.8) | (0.1, 0.5) |
| othe rpets | 21 | 3 | 41 | 7 | 0.8 | (0.2, 3.5) | (0.3, 16.2) |
| pets at home | 21 | 19 | 46 | 39 | 1.7 | (0.3, 9.0) | (0.1, 4.9) |
| pets outside | 21 | 7 | 66 | 18 | 1.3 | (0.5, 3.8) | (0.6, 5.5) |
| pork | 21 | 4 | 66 | 46 | 0.1 | (0.0, 0.3) | (0.0, 0.2) |
| pork sausage | 21 | 5 | 57 | 23 | 0.5 | (0.1, 1.4) | (0.2, 1.1) |
| poultry | 21 | 10 | 70 | 53 | 0.3 | (0.1, 0.8) | (0.1, 0.7) |
| reptile | 21 | 14 | 41 | 3 | 25.3 | (5.7, 111.8) | (5.5, 265.2) |
| salad | 21 | 5 | 60 | 40 | 0.2 | (0.1, 0.5) | (0.1, 0.3) |
| steak | 21 | 1 | 48 | 8 | 0.3 | (0.0, 2.1) | (0.1, 4.9) |
